# Neurotrophic and synaptic effects of GnRH and/or GH upon motor function after spinal cord injury in rats

**DOI:** 10.1038/s41598-024-78073-3

**Published:** 2024-11-02

**Authors:** C. G. Martínez-Moreno, D. Calderón-Vallejo, C. Díaz-Galindo, I. Hernández-Jasso, J. D. Olivares-Hernández, J. Ávila-Mendoza, D. Epardo, J. E. Balderas-Márquez, V. A. Urban-Sosa, R. Baltazar-Lara, M. Carranza, M. Luna, C. Arámburo, J. L. Quintanar

**Affiliations:** 1 Departamento de Neurobiología Celular y Molecular, Instituto de Neurobiología, Universidad Nacional Autónoma de México, Campus Juriquilla, Querétaro, México; 2 Departamento de Fisiología y Farmacología, Centro de Ciencias Básicas, Universidad Autónoma de Aguascalientes, Aguascalientes, México

**Keywords:** Spinal cord injury, Gonadotropin-Releasing Hormone, Growth Hormone, Neurotrophins, Synaptogenesis, Neuroprotection, Spinal cord injury, Spine plasticity

## Abstract

Thoracic spinal cord injury (SCI) profoundly impairs motor and sensory functions, significantly reducing life quality without currently available effective treatments for neuroprotection or full functional regeneration. This study investigated the neurotrophic and synaptic recovery potential of gonadotropin-releasing hormone (GnRH) and growth hormone (GH) treatments in ovariectomized rats subjected to thoracic SCI. Employing a multidisciplinary approach, we evaluated the effects of these hormones upon gene expression of classical neurotrophins (NGF, BDNF, and NT3) as well as indicative markers of synaptic function (Nlgn1, Nxn1, SNAP25, SYP, and syntaxin-1), together with morphological assessments of myelin sheath integrity (Klüver-Barrera staining and MBP immunoreactivity) and synaptogenic proteins (PSD95, SYP) by immunohystochemistry (IHC) , and also on the neuromotor functional recovery of hindlimbs in the lesioned animals. Results demonstrated that chronic administration of GnRH and GH induced notable upregulation in the expression of several neurotrophic and synaptogenic activity genes. Additionally, the treatment showed a significant impact on the restoration of functional synaptic markers and myelin integrity. Intriguingly, while individual GnRH application induced certain recovery benefits, the combined treatment with GH appeared to inhibit neuromotor recovery, suggesting a complex interplay in hormonal regulation post-SCI. GnRH and GH are bioactive and participate in modulating neurotrophic responses and synaptic restoration under neural damage conditions, offering insights into novel therapeutic approaches for SCI. However, the intricate effects of combined hormonal treatment accentuate the necessity for further investigation that conduce to optimal and novel therapeutic strategies for patients with spinal cord lesions.

## Introduction

Spinal cord trauma at the thoracic level produces significant detrimental motor and sensory outcomes, affecting particularly hindlimb and urinary general functions. Patients with thoracic spinal cord injury (SCI) experience a severe decline in overall health and life quality^1,2^. Unfortunately, to date, the availability of effective neuroprotective clinical therapies that promote functional regeneration in the damaged spinal cord tissue is still scarce, and do not provide full recovery of sensitivity and movement. Understanding the cellular and molecular interactions triggered after a mechanical neural injury is crucial to modulate the intense stress on metabolism and an exacerbated inflammatory and immune responses^2^. During the acute stage of SCI, there is a rapid cellular infiltration of macrophages and neutrophils, which plays a critical role in the healing process, given the dependence of the beneficial M2/A2 phenotype of microglia/astrocytes on these interactions^3–5^. The inflammatory process initiates a trophic intra- and extracellular signaling cascade, that takes control over tissue regenerative and healing mechanisms^5^. Within the nervous system, the neurotrophic storm begins just minutes after trauma and includes a response that involves the biological activity of classical neurotrophins, such as brain-derived neurotrophic factor (BDNF), nerve growth factor (NGF), and neurotrophin-3 (NT3)^6–9^. However, until now, no information was available about the modulatory effect of growth hormone (GH) or gonadotropin-releasing hormone (GnRH) upon the expression of classical neurotrophins and synaptic/neural recovery markers.

In recent years, there has been a notable increase in reports concerning the novel neurotrophic actions of classical hormones, polypeptides, and growth factors, each associated with distinct canonical functions such as somatic growth and reproduction. Notably, GH and GnRH have been found to exert actions within the nervous system that promote cell survival through anti-apoptotic effects, anti-inflammatory actions, neurite branching, axonal growth, and potentially functional synaptogenesis^10^. The anti-inflammatory actions of GH and GnRH have been found to be highly effective in the SCI compressive model, particularly in the tissue located in the caudal portion of the injury site and both hormones were able to restore sensorial function, as assessed by the hotplate test^11^.

The effect of GnRH treatment on the recovery of extremities functionality post-thoracic SCI has been evidenced in both animal models and patients^10–13^. However, the investigation regarding the molecular and cellular mechanisms involved in the neurotrophic activities of this hypothalamic factor has been limited until now. Much remains to be understood about the non-canonical actions of GnRH, particularly due to the extensive expression of its receptor, GnRHR, in the nervous system and various peripheral tissues^11^. The presence of the GnRH receptor in the spinal cord has been documented in animal models and humans, indicating its potential roles within the SCI physiopathology and in other neurological disorders^14–16^. GnRHR is a G-protein-coupled receptor that activates protein kinase C, which is associated with various survival effects in the central nervous system (CNS) during development^17^. The exact interactive implications (v.gr. agonism, antagonism or interference) of simultaneous activation of the GnRH receptor and other receptor types, remain unknown. Meanwhile, the actions of GH in the nervous system extend beyond growth and development. Research focused on the application of GH in patients with brain trauma and other neurodegenerative diseases has been increasing in recent years^18^. The activation of the JAK/STAT pathway and the anti-apoptotic Akt pathway are outcomes of GH binding to its mature and functional receptor on the cell surface, thus inducing the expression of survival genes, including neural factors like BDNF and NT3^19,20^. GHR gene is expressed in the spinal cord, particularly during development and growth, in various species such as humans, rats, bovines, chicks, and zebrafish^21–23^.

This work presents an analysis of gene expression for markers associated with synaptic function such as neuroligin-1 (Nlgn1), neurexin-1 (Nrxn1), SNAP-25, syntaxin-1 and synaptophysin (SYP) as well as classical neurotrophins (NGF, BDNF, NT3) in ovariectomized (OVX) animals -to specifically avoid the influence of estrogen neuroprotection- that were subjected to SCI and treated with either GnRH, GH, or the combined treatment. Our study also included an evaluation of myelin sheath integrity using Klüver-Barrera staining and IHC, as well as immunohistochemical observations for NT3-, CNTF-, PSD95-, and SYP-immunoreactivities (IR). In addition, we incorporated an assessment of neuromotor recovery based on the Basso, Beattie, and Bresnahan (BBB) scale determination and ankle kinematics^12^.

## Results

### hGH and GnRH upregulate neurotrophic gene expression

**Fig. 1:**
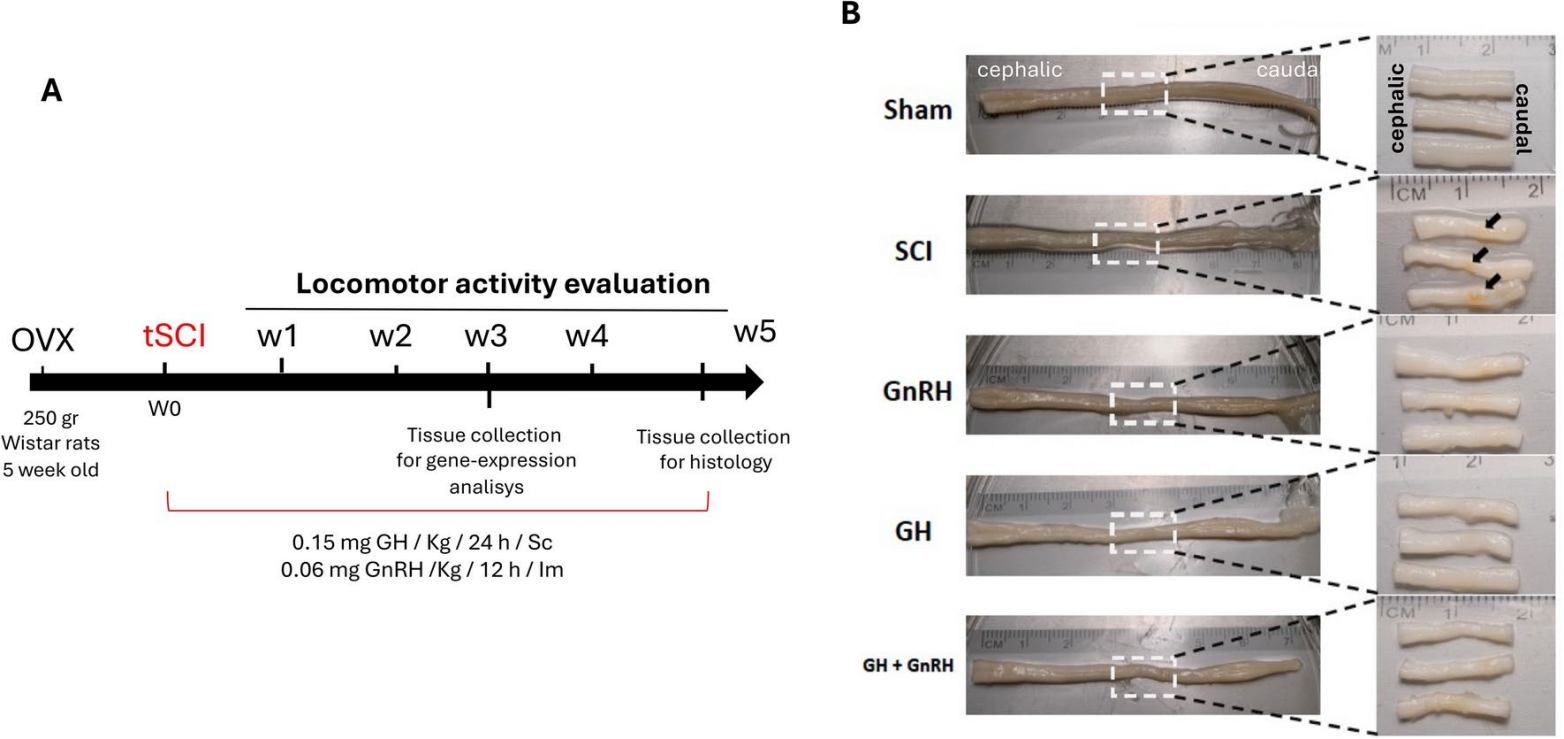
**A** Schematic representation of the experimental design and timeline. **B** Spinal cord samples obtained from OVX rats at week 5 post-injury. Inserts show 1.5 cm tissue pieces that were obtained. Arrows show damage (yellow-orange) in the spinal cord tissue. Cephalic and caudal sections are included for anatomic orientation. Sham group control with surgery and without compressive damage by catheter insufflation.

After 5 weeks of treatment, the animals’ body weight was not affected (Sup. Figure 1A). However, the body length was significantly increased (P < 0.05) by GH and GnRH, but not in combination (Sup. Figure 1B). The full spinal cord, measuring between 7–8 cm, was extricated from the vertebral canal by injecting water with a syringe. A section measuring 1.5 cm using the injury epicenter (T10) as the middle point, was excised for subsequent biochemical and molecular analyses. The spinal cord sectioning was performed in wet tissue right after the pressure ejection. Macroscopic evaluation of the SCI displayed a pronounced reduction in spinal cord diameter accompanied by the emergence of a yellowish stain at the lesion’s epicenter (denoted by arrows). These colored regions are attributable to the compression injury brought on by the catheter insufflation and are particularly pronounced in the SCI group (Fig. 1B).

**Fig. 2:**
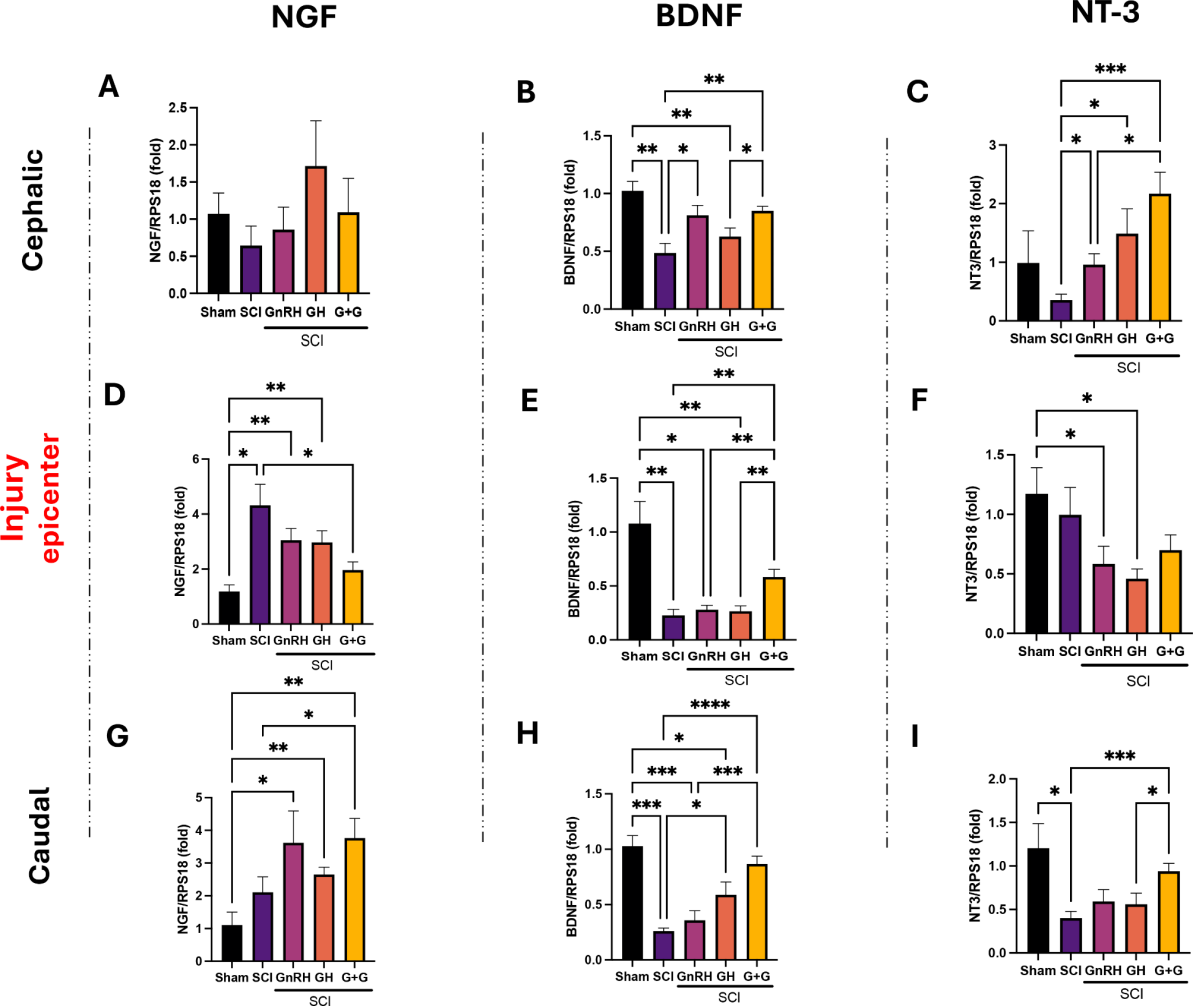
Relative gene-expression of NGF, BDNF and NGF (neurotrophins) in the lesioned spinal cord tissue. **A, D, G**: relative change of NGF mRNA; **B, E, H**: relative change of BDNF mRNA; **C, F, I**: relative change of NT3 mRNA. Spinal cord section analyzed (relative to T10): **A–C**: cephalic (rostral) – T9-T8; **D–F**: injury site (epicenter)—T10-T11; **G–I**: caudal (distal)—T12-T13. Experimental groups: sham (control), spinal cord injury (SCI), GnRH, GH and GnRH + GH (G + G). RPS18: reference gene. Values are represented as mean ± SEM. At least 5 animals are included per group from 2 independent experiments. One-way Brown-Forsythe and Welch ANOVA with Fisher´s LSD *post-hoc* test identified significant differences. A p-value under 0.05 indicated statistical significance and is represented with asterisks as follow: * P < 0.05, ** P < 0.01, *** P < 0.001 and **** P < 0.0001.

Gene expression assessments using qPCR focused on classical neurotrophins: NGF, BDNF, and NT3 (Fig. 2). Data indicated that tissue samples obtained from the cephalic (head side) region consistently manifested responses to all the treatments evaluated in this study. Injury did elevate NGF expression in tissue sampled from the damage epicenter (P < 0.05; Fig. 2D) and, in contrast, the combination of GH and GnRH resulted in its downregulation (P < 0.05); it is important to note that individual treatments abolished the statistical difference (P < 0.05) induced by damage despite there was no significance when compared to SCI group. Notably, treatments did not show significant changes in NGF gene expression in the cephalic segment (Fig. 2A) of the spinal cord. It was also remarkable that NGF was the only neurotrophin that significantly increased its expression by SCI (Fig. 2D). For tissue obtained from the caudal portion, damage by itself did not significantly alter NGF gene expression when compared with the sham control, but there was a marked surge in NGF expression in both the GnRH (P < 0.05) and the combined groups (P < 0.01) relative exclusively to the undamaged group; however a significant difference was also observed in the G + G experimental group when compared to the SCI group (P < 0.05). On the other hand, in comparison with the control SCI significantly attenuated BDNF gene expression across all three spinal tissue segments assessed (see Figs. 2B, 2E, 2H). In the cephalic segment (Fig. 2B), injury decreased BDNF mRNA expression (P < 0.05) whereas the GnRH treatment, either administered alone (P < 0.05) or in combination (P < 0.01), significantly increased it in relation with the SCI group. At the injury epicenter (Fig. 2E), the lesion drastically reduced the expression of BDNF mRNA in the following groups as compared to the sham control without neural damage: SCI (P < 0.01), GnRH (P < 0.05) and GH (P < 0.01). However, in this section, the spinal tissue from animals treated with G + G revealed significantly (P < 0.01) elevated levels (Fig. 2E) in comparison to the SCI group. Within the caudal segment (Fig. 2H), the samples receiving GH treatment, either alone or combined with GnRH, showed a substantially greater BDNF mRNA expression as compared to the SCI group (P < 0.05 and P < 0.001, respectively). Lastly, NT3 gene expression remained unaffected by compressive damage in the rostral section and epicenter, showing tendency to decrease but without significant difference (Figs. 2C, 2F) in comparison to the controls. In contrast, the caudal section showed a significant decrease induced by SCI (Fig. 2I; P < 0.05). Interestingly, in the cephalic section, GnRH and GH, when administered separately, slightly upregulated NT3 gene expression (P < 0.05), in comparison to the lesioned group, whereas their combination led to a notable upregulation (P < 0.001) in transcriptional activity in comparison to the SCI condition. In the injury site, we found that GnRH and GH in damaged animals reduced (P < 0.05) the expression of NT3 when compared to animals without neural damage but no difference was observed in relation to the SCI group. Tissue collected from the caudal side of the injury, showed a strong effect upon NT3 mRNA expression when G + G was applied to lesioned animals, although neither one of the hormones, when administered alone exerted any effect.

### hGH and GnRH enhance the expression of synaptic vesicle activity markers

**Fig. 3:**
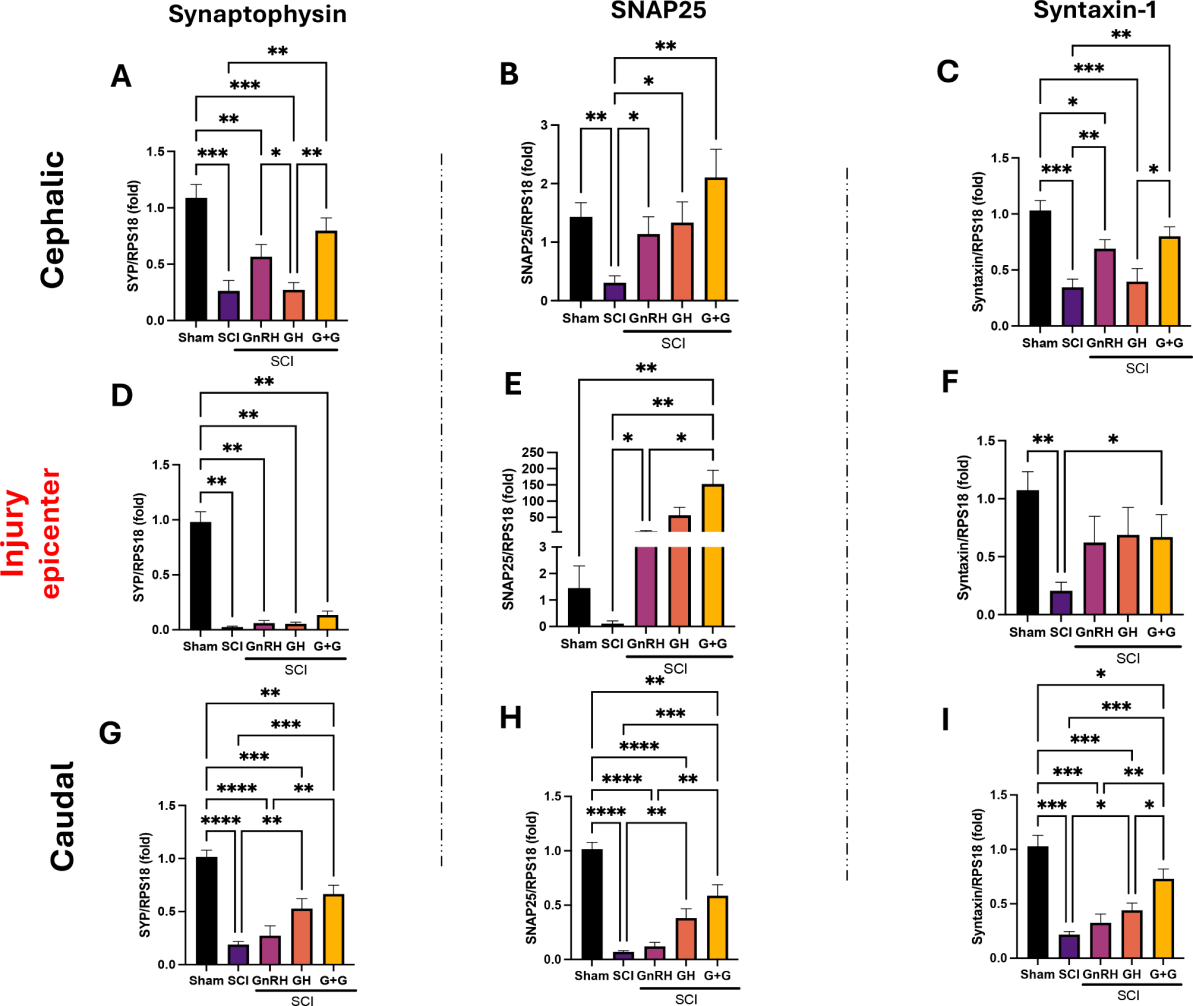
Relative gene-expression of SYP, SNAP25 and Syntaxin-1 (SNARE complex elements) in the lesioned spinal cord tissue. **A, D, G**: relative change of SYP mRNA; **B, E, H**: relative change of SNAP25 mRNA; **C, F, I**: relative change of syntaxin-1 mRNA. Spinal cord section analyzed (relative to T10): **A-C**: cephalic (rostral) – T9-T8; **D-F**: injury site (epicenter)—T10-T11; **G-I**: caudal (distal)—T12-T13. Experimental groups: sham (control), spinal cord injury (SCI), GnRH, GH and GnRH + GH (G + G). RPS18: reference gene. Values are represented as mean ± SEM. At least 5 animals are included per group from 2 independent experiments. One-way Brown-Forsythe and Welch ANOVA with Fisher´s LSD *post-hoc* test identified significant differences. A p-value under 0.05 indicated statistical significance and is represented with asterisks as follow: * P < 0.05, ** P < 0.01, *** P < 0.001 and **** P < 0.0001.

The gene expression of SYP, SNAP25, and syntaxin-1, indicative of synaptic vesicle activity, was investigated in spinal cord tissue from the T10 region following SCI (Fig. 3). For SYP mRNA expression, a substantial reduction was observed across all spinal segments in response to SCI (Figs. 3A, 3D, 3G). In the cephalic portion, there was a marked decrease in SYP expression (P < 0.001) after injury. The combined GnRH + GH combined treatment reverted transcriptional levels to those seen in the undamaged group and showed a significant difference (P < 0.01) when compared to the SCI group. At the injury site, SCI significantly reduced SYP transcription (P < 0.01), with no treatment being effective to counteract this strong effect. In the caudal portion, SCI notably downregulated SYP transcripts (P < 0.0001), which were partially reversed by GH treatment alone (P < 0.01) and further improved with the combined GnRH + GH treatment (P < 0.001), although not fully restored. In turn, regarding SNAP25 mRNA expression, a significant downregulation (P < 0.01) was observed in the cephalic portion after SCI, which was reversed with either GH (P < 0.05) or GnRH (P < 0.05) treatment (Fig. 3B), whereas the combined treatment showed a stronger effect as compared to the SCI group (P < 0.01), restoring transcriptional rates to levels akin to the sham control. At the injury site (Fig. 3E), treatments elicited a greater amplified response. While SCI alone did not significantly differ from the sham control, GnRH treatment alone increased SNAP25 mRNA levels over 40-fold, and the combined treatment led to an approximately 150-fold increase (P < 0.01). GH alone also increased levels about 50-fold, but this was statistically distinct from the SCI group only when compared by t-test (P < 0.05). In the caudal region (Fig. 3H), both GH treatment and the combined GH + GnRH administration led to a partial recovery of SNAP25 mRNA levels (P < 0.01), albeit less than in other spinal sections. On the other hand, syntaxin-1 expression also demonstrated a significant downregulation throughout the lesioned spinal tissues (Figs. 3C, 3F, 3I). In the cephalic region, SCI reduced syntaxin-1 mRNA by ~ 60% (P < 0.001), with GnRH alone or in combination with GH partially restoring its transcriptional rates (P < 0.01). At the injury site (Fig. 3F), only the combined treatment differed significantly from the SCI group, despite similar mean effects in independent GnRH and GH treatments. In the distal portion (Fig. 3I), SCI reduced syntaxin-1 expression by ~ 60%, with GH treatment alone showing a significant difference (P < 0.05) and the combined treatment having a stronger effect (P < 0.001). It is important to note that in the caudal section, a similar response pattern to treatments was observed for all studied genes (SYP, SNAP25, and syntaxin-1), reflecting a consistent but partial recovery across these synaptic markers.

### hGH and GnRH restore the expression of functional synaptic markers

**Fig. 4:**
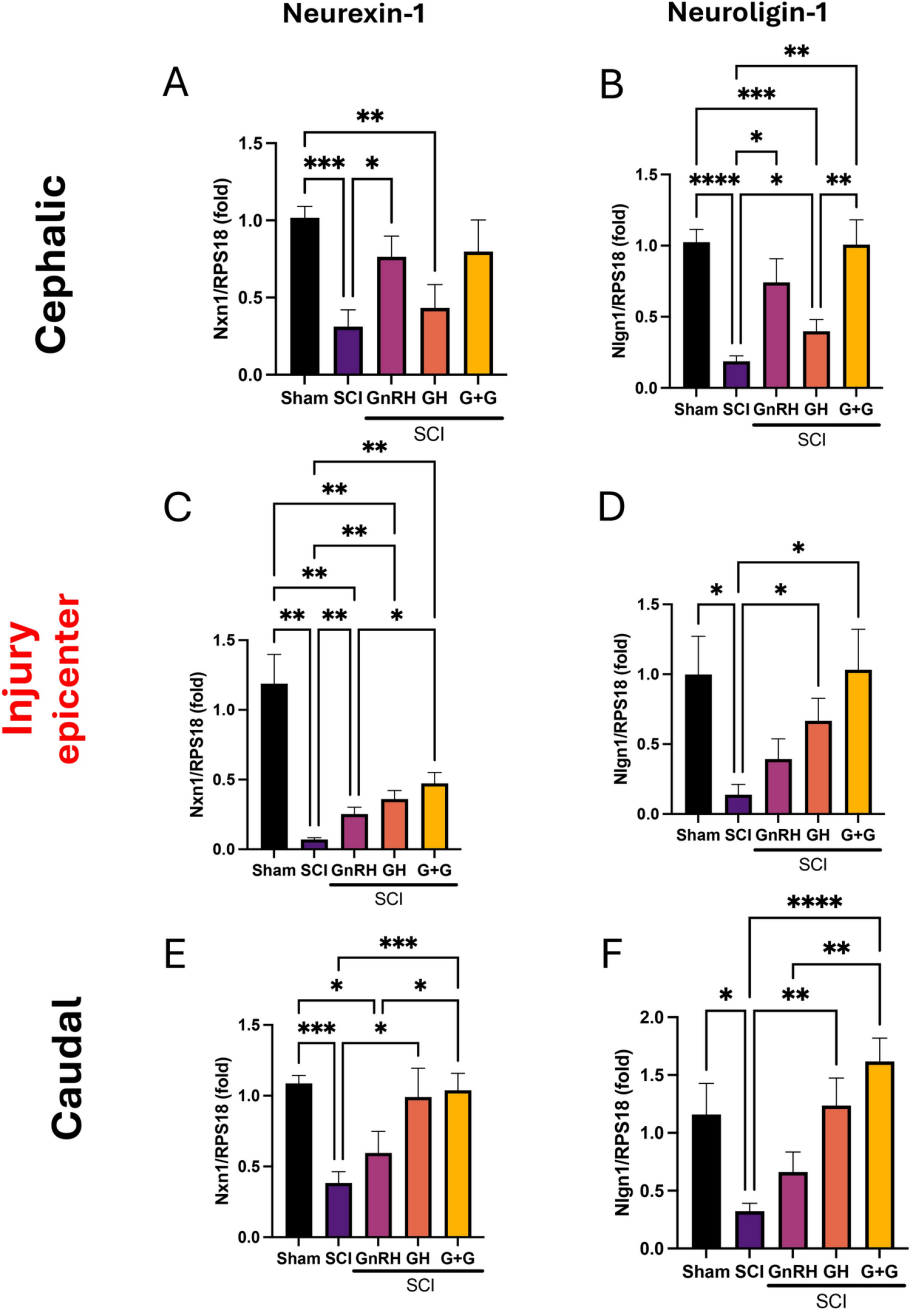
Relative gene-expression of neurexin-1 (Nxn1) and neuroligin-1 (Nlgn1) (functional synaptic markers) in the lesioned spinal cord tissue. **A, C, E**: relative change of Nxn1 mRNA; **B, D, F**: relative change of Nlgn1 mRNA. Spinal cord section analyzed (relative to T10): **A, B**: cephalic (rostral) – T9-T8; **C, D**: injury site (epicenter)—T10-T11; **E, F**: caudal (distal)—T12-T13. Experimental groups: sham (control), spinal cord injury (SCI), GnRH, GH and GnRH + GH (G + G). RPS18: reference gene. Values are represented as mean ± SEM. At least 5 animals are included per group from 2 independent experiments. One-way Brown-Forsythe and Welch ANOVA with Fisher´s LSD *post-hoc* test identified significant differences. A p-value under 0.05 indicated statistical significance and is represented with asterisks as follow: * P < 0.05, ** P < 0.01, *** P < 0.001 and **** P < 0.0001.

Following SCI, an extensive decrease in Nrxn-1 mRNA levels within the spinal cord sections was observed (Figs. 4A, 4C, 4E), indicating a significant loss of synaptic function. In the cephalic portion, there was a marked reduction in Nrxn-1 expression (P < 0.001); however, restoration of these levels to those similar to the controls was achieved exclusively with GnRH treatment (P < 0.05), while GH showed no effect. Although the combined GnRH + GH treatment mirrored the response pattern of GnRH alone, it did not yield a statistically significant difference compared to the SCI group in this tissue section (Fig. 4A). At the damage epicenter, a marked decrease in Nrxn-1 gene expression was noted (P < 0.01). All treatments induced a moderate yet significant response (P < 0.01), with the combined GnRH and GH (G + G) treatment exhibiting the most pronounced effect, achieving the highest mean among the experimental groups. This suggests that while individual treatments had some efficacy, the combined treatment was more effective in enhancing Nrxn-1 expression (Fig. 4C). In the distal portion, there was a significant decrease in Nrxn-1 mRNA levels (P < 0.05) provoked by SCI. This reduction was completely reversed by GH (P < 0.01) and the combined G + G treatment (P < 0.0001) in comparison to the damaged group (Fig. 4E). Notably, the combined treatment of GnRH and GH was the only treatment that reestablished Nrxn-1 gene expression in all three analyzed sections, highlighting its efficacy in synaptic function recovery post-SCI. On the other hand, Nlgn1 gene expression was diminished by injury in the three sections of the spinal cord analyzed (Figs. 4B, 4D, 4F). In the rostral section, we observed a restorative effect of GnRH (P < 0.05), GH (P < 0.05), and G + G (P < 0.01) treatments, indicating a statistically significant difference when compared with the untreated SCI group (Fig. 4B). In the injury site, GH alone and combined with GnRH was effective in restoring Nlgn1 gene expression (Fig. 4D). In the caudal section, GH alone completely restored Nlgn1 gene expression, and its combination with GnRH showed an even higher statistical difference (P < 0.0001) when compared to the damaged group.

### Effects of hGH and GnRH on Myelin sheath integrity as determined by Klüver-Barrera Staining and MBP immunoreactivity

**Fig. 5:**
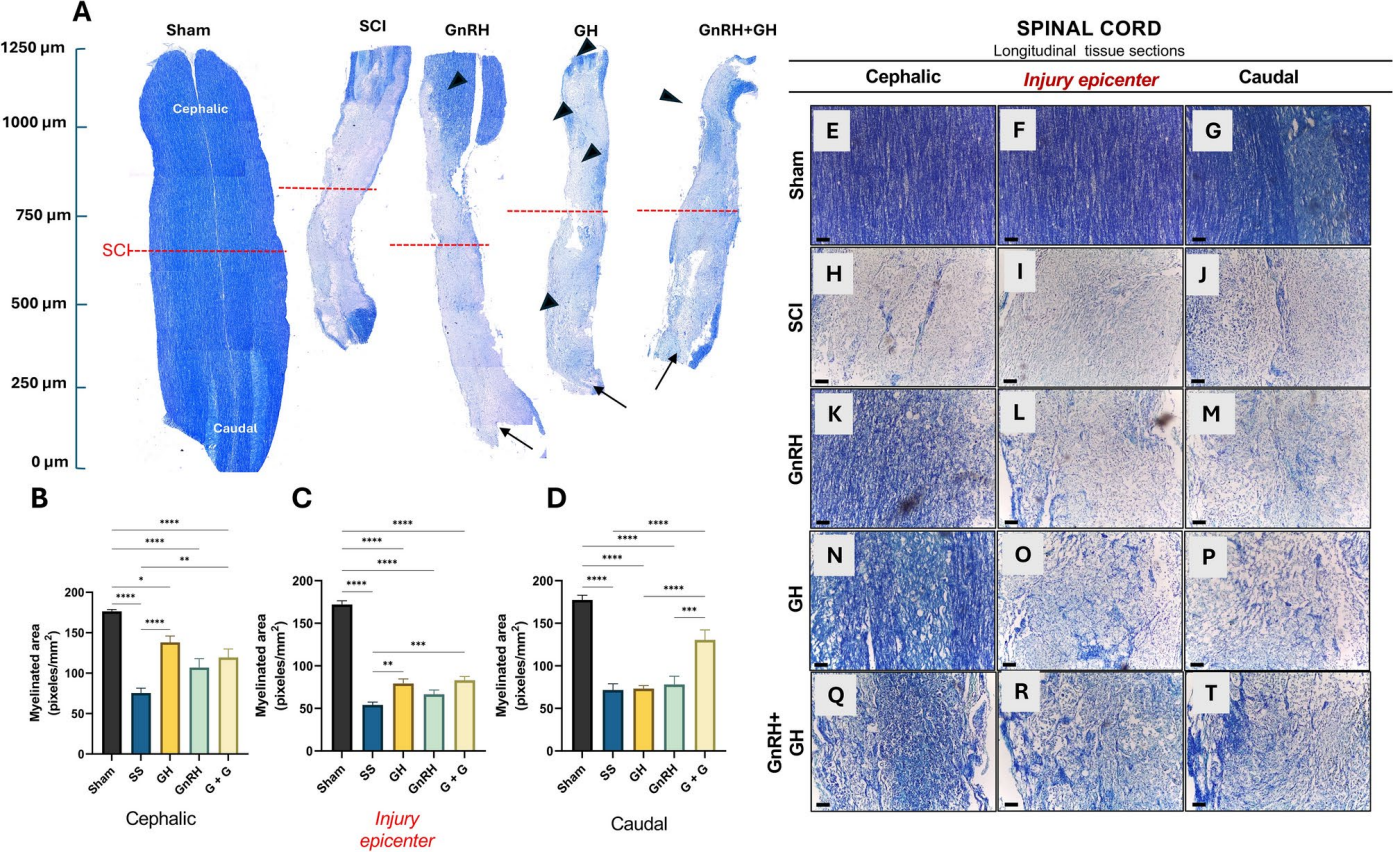
Myelin integrity analysis by Klüver-Barrera staining in longitudinal sections of spinal cord with thoracic damage. (**A**) panoramic images of 1.5 cm sections of spinal cord tissue. (**B–D**) Myelin semi-quantification. Representative images of each experimental group with positive (blue) staining: (**E–G**) sham (control), (**H–J**) spinal cord injury (SCI), GnRH (**K–M**), GH (**N-P**) and GnRH + GH (G + G; **Q-T**). Scale bar: 20 µM. Values are represented as mean ± SEM. Tissues from 3 animals were analyzed in every portion of the spinal cord: cephalic, injury epicenter and caudal. One-way Brown-Forsythe and Welch ANOVA with Fisher´s LSD *post-hoc* test identified significant differences. A p-value under 0.05 indicated statistical significance and is represented with asterisks as follow: * P < 0.05, ** P < 0.01, *** P < 0.001 and **** P < 0.0001. Units are in microns (µm).

Figure 5A shows representative panoramic micrographs from longitudinal sections of spinal cord tissues stained with Klüver-Barrera to assess myelin sheath integrity. It was evident that, in comparison to the sham controls, the lesioned tissues had a differential response to dehydration during the histological processing, showing a strong shrinking effect that substantially modified the length and width size of the fixed tissues. This was partially prevented with GnRH treatment (Fig. 5A). For orientation matters, the red dotted line shows the injury epicenter (Fig. 5A). The tissue from the sham group displayed an intact spinal cord section with densely stained myelin fibers in the ventral white matter. Lighter staining in the caudal region highlights the posterior horns (arrows). Conversely, spinal cord slices from SCI animals without hormonal treatment (Fig. 5A) showed reduced spinal cord size as mentioned above, and Klüver-Barrera staining was faint, confined mainly to the tissue poles. The central part of the SCI sample appeared lighter in staining and disorganized. Interestingly, GnRH treatment positively influenced the size of the spinal cords, yet signs of compressive damage persisted, as evidenced by the predominant loss of staining. Nonetheless, there was an uptick in staining at the cephalic pole (arrowheads) and an overall improvement in cytostructural organization. GH treatment resulted in myelin recovery at the poles, as well as a general increase in Klüver-Barrera staining in various spots (arrowheads), though it didn’t match the size recovery observed with GnRH. The combination of GnRH and GH led to a significant increase in overall Klüver-Barrera staining intensity.

A semi-quantitative densitometric analysis of the blue staining (indicative of myelin sheath integrity) was conducted using Fiji software to measure blue intensity. Due to tissue disorganization, areas for analysis were selectively chosen based on positive myelin staining. This revealed a differential pattern of damage, with partial recovery by the treatments. In the cephalic portion of the injured spinal cord, GH and the combined treatment significantly enhanced the blue color intensity (pixels per mm^2^) in selected fields (P < 0.0001 and P < 0.01, respectively), whereas GnRH also tended to increase staining, though not significantly (Fig. 5B). At the injury epicenter, both GH and the combined treatment significantly intensified Klüver-Barrera staining (P < 0.01 and P < 0.0001, respectively), but with a lower intensity compared to the rostral end (Fig. 5C). In the caudal section from lesioned rats treated with the GnRH and GH combination, there was an increase in myelin staining, whereas GH and GnRH alone showed no effects (Fig. 5D).

**Fig. 6:**
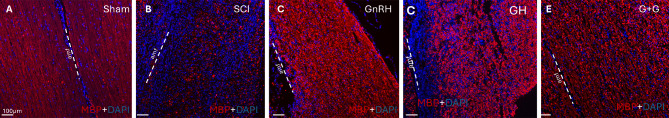
Immunohystochemistry (IHC) for myelin basic protein** (**MBP) in spinal cord tissue. Longitudinal sections of spinal cord collected from epicenter of the injury (T10). Experimental groups: (**A**) sham (control), (**B**) spinal cord injury (SCI), (**C**) GnRH, (**D**) GH and (**E**) GnRH + GH (G + G). Red: MPB immunoreactivity; Blue: DAPI staining (nuclei). Scale bar (50 µm).

Microscopic imaging revealed that the sham group had a high staining intensity with well-organized parallel fibers (Figs. 5E, 5F, 5G), whereas the SCI group exhibited very low staining in all three analyzed tissue sections (Figs. 5H, 5I, 5J). In the GnRH-treated group, tissue appeared better organized with mild blue intensity (Figs. 5K, 6L, 6M). Intriguingly, GH treatment markedly increased Klüver-Barrera staining in fibrillar structures, albeit without an organized pattern (Figs. 5N, 5O, 5P). This effect of enhanced blue staining in disorganized structures was more evident in images showing combined GnRH and GH treatment (Figs. 5Q, 5R, 5S). Interestingly, a higher myelin staining was observed in the cephalic sections with all treatments in comparison to the other regions (Figs. 5K, 5N, 5R).

<?float p8,top?>In addition, we performed myelin basic protein (MBP) IHC to assess myelin sheath recovery in longitudinal tissue sections. In the sham group, we observed well-organized fibers with positive MBP-IR (Fig. 6A). However, after SCI, the fibers became completely disorganized, and a marked reduction in MBP-IR was evident (Fig. 6B). Treatment with GnRH resulted in a significant recovery of tissue organization, along with a robust increase in MBP-IR (Fig. 6C). In turn, GH treatment led to an increase in MBP immunofluorescence; but the tissue organization did not appear to improve post-SCI. Interestingly, the combined treatment group (G + G) exhibited better tissue organization than the GH-treated group although showed slightly lower MBP fluorescence compared to the individual treatments with either GH or GnRH.

### Effects of GnRH and GH on III-β-tubulin immunoreactivity in SCI

**Fig. 7:**
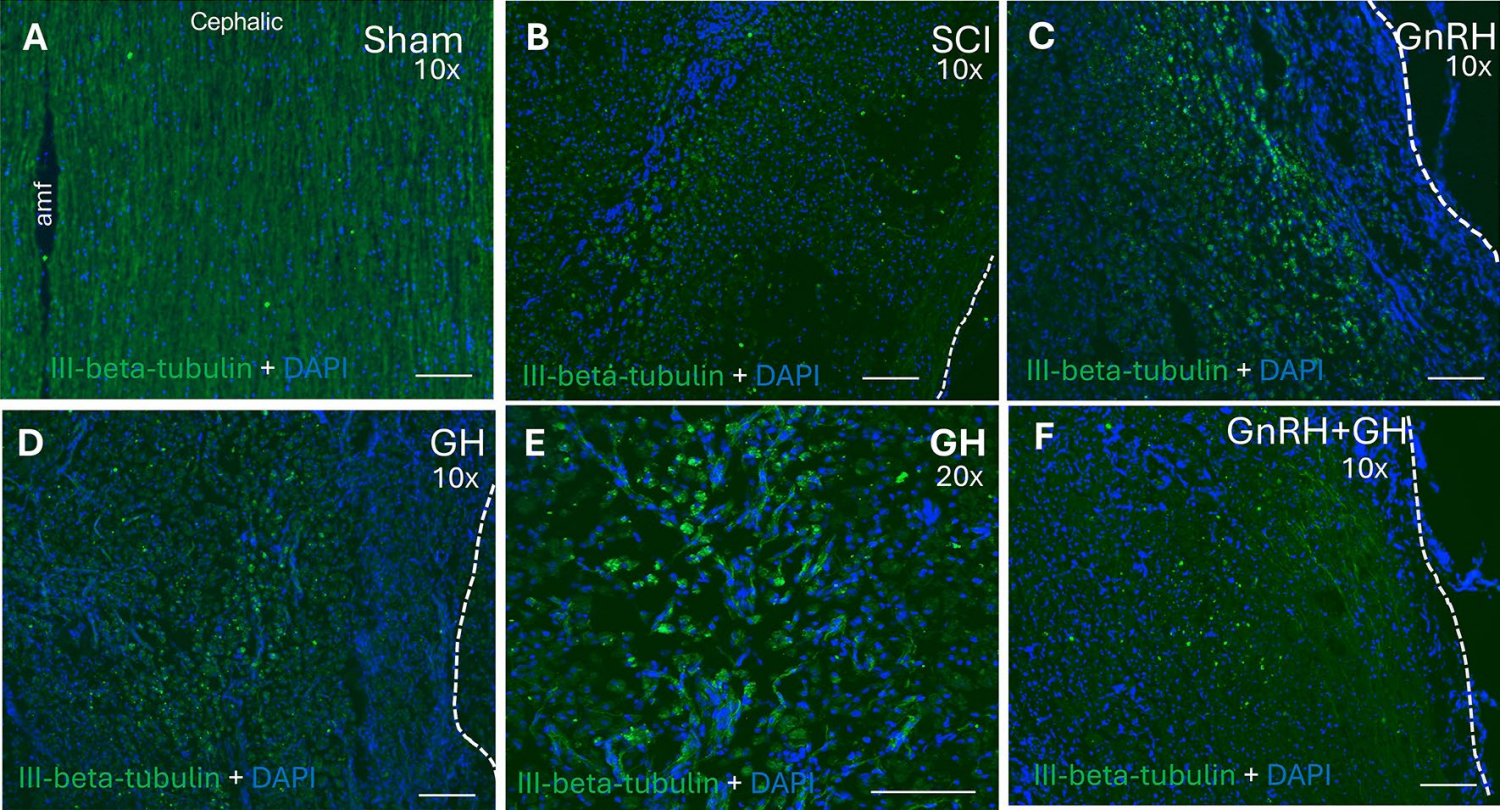
III-beta-tubulin immunoreactivity in spinal cord tissue. Longitudinal sections of spinal cord collected from epicenter of the injury (T10). Experimental groups: (**A**) sham (control) at 10x, (**B**) spinal cord injury without treatment (SCI), (**C**) GnRH, (**D**) GH at 10 × and (**E**) GH at 20 × and (**F**) GnRH + GH (G + G). Green: III-B-tubulin immunofluorescence; Blue: DAPI staining (nuclei). Scale bar (50 µm).

In longitudinal sections of the spinal cord, positive staining with well-organized fibers corresponding to the ventral white matter was evident (Fig. 7A) in the controls. The image shows the anterior median fissure (amf) and DAPI-positive nuclei from cells along the immunoreactive fibers. Damage leaded to a significant reduction in III-β-tubulin and caused complete disruption of the spinal cord’s fiber arrangement, with a sparse presence of III-β-tubulin IR-positive cells; however, these cells were not specifically identified in this study (Fig. 7B). In lesioned animals treated with GnRH, the tissue exhibited a healing pattern, as III-β-tubulin positive cells tended to form a columnar organization with intense immunofluorescence (Fig. 7C). On the other hand, GH had a distinct effect since positive cells for III-β-tubulin displayed an aberrant pattern in the spinal cord (Figs. 7D and 7E). Interestingly, the combination of GnRH and GH revealed a strong resemblance to the SCI group (Fig. 7F), characterized by very low III-β-tubulin immunofluorescence and the accumulation of cells at the center of the injury.

### Effects of GnRH and GH on NT3 immunoreactivity in SCI

**Fig. 8:**
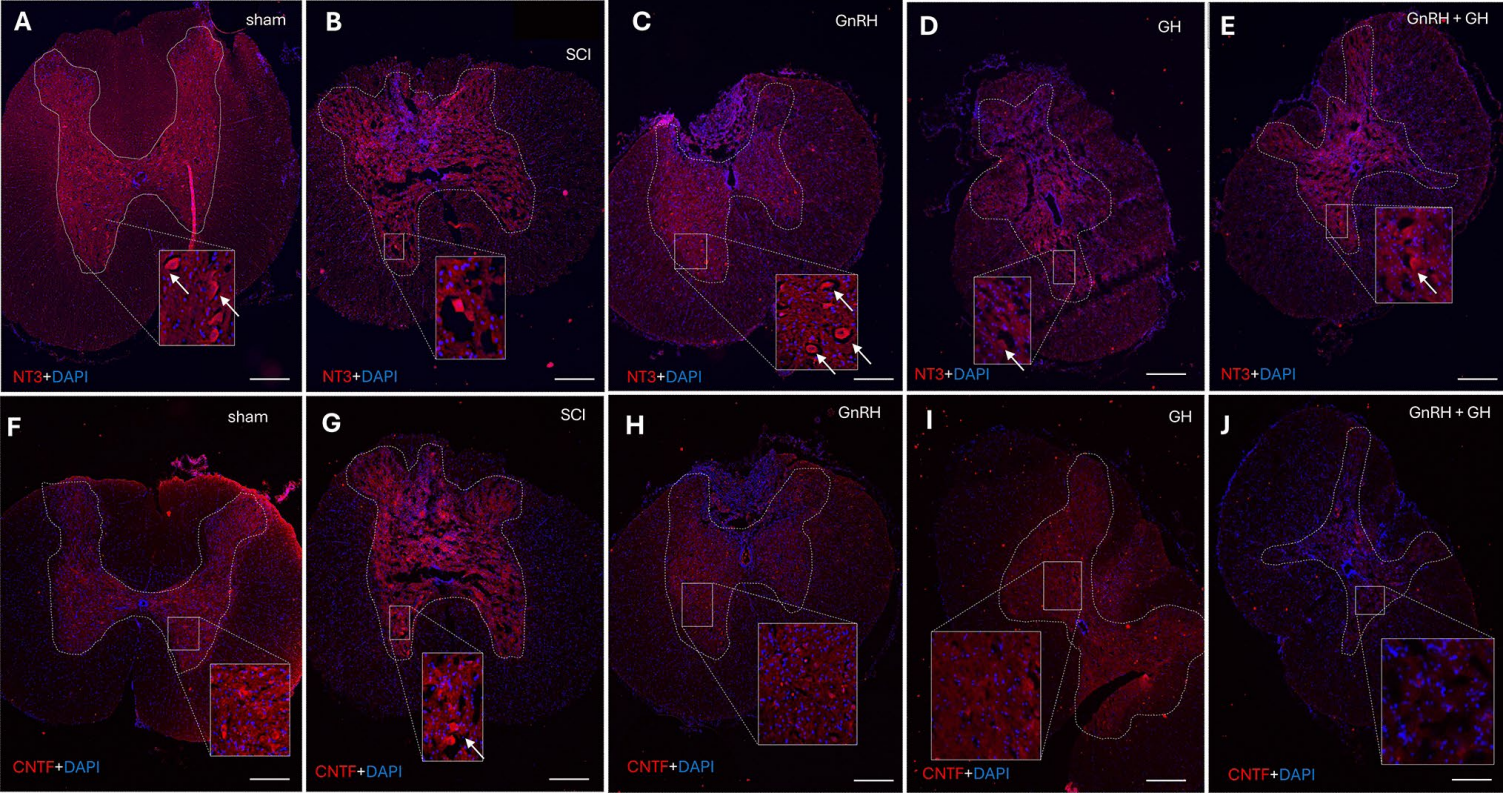
NT3 and CNTF immunohistochemistry in spinal cord tissue. Transverse sections of spinal cord collected from distal side of the injury (T11-T12). NT3-immunofluorescence (red) at 4 × magnification (**A-E**) and CNTF-immunofluorescence (red) at 4 × magnification (**F-J**). DAPI was used for nuclei counterstaining (blue). Arrows show representative immunopositive cells. Experimental groups: sham (control), spinal cord injury (SCI), GnRH, GH and GnRH + GH (G + G). Scale bar (100 µm). Inserts are a digital amplification of representative areas in the dorsal horns of the spinal cord.

We conducted a study using 15 µm transversal sections from the spinal cord, particularly from the rostral area of the injury. These sections were subjected to cryosectioning and then incubated with an NT3-specific antibody for detailed IHC examination. In spinal cords from control rats, we observed a pronounced NT3 immunofluorescence in the motor neuron cell bodies, indicated by arrows in the insert, located in the gray matter’s dorsal and ventral horns (Fig. 8A). However, in samples derived from lesioned animals (SCI), the gray matter exhibited a significant disarray, with apparent gaps that likely indicate tissue loss (Fig. 8B). This damage was accompanied by numerous small DAPI-positive cells infiltrating the dorsal end. Upon examining the effects of GnRH treatment (Fig. 8C), we noted a distinct improvement in tissue organization and the distribution of white and gray matter. Motor neurons resembling those in the control group’s tissue, both in shape and NT3 reactivity, were identifiable. Specifically, in the GnRH-treated animals, the spinal cord tissue exhibited clusters of DAPI-positive cells in the white matter’s dorsal columns. These cells also showed mild NT3 immunofluorescence (Fig. 8C; arrowheads). Conversely, the group treated with GH displayed no noticeable changes in cell integrity or NT3 staining (Fig. 8D). Lastly, tissue sections from animals treated with both hormones resembled those of the SCI group in terms of structure. However, they lacked the infiltrated cells typically present in untreated damaged tissue (Fig. 8E).

### Effect of GnRH and GH on CNTF immunoreactivity in SCI

We also investigated the presence of CNTF-IR in the tissue samples, which showed irregular increases in the gray matter due to SCI, indicating significant tissue disruption and the absence of distinct CNTF-positive motor neurons (Fig. 8G). The exact cell type synthesizing this neurotrophic factor remains unclear. In the control group, positive staining was predominantly observed in neurons situated in the ventral horns (Fig. 8F), which are either missing or morphologically altered due to damage. Interestingly, chronic treatment with GnRH had a noticeable impact on tissue structure. Positive neurons for CNTF were observed in the ventral horns of the gray matter (Fig. 8H). Notably, in the GnRH-treated group, there was an increase in the number of DAPI-positive nuclei, particularly in the area corresponding to the sensory dorsal columns. On the other hand, GH treatment seemed to restore CNTF-IR, with identifiable CNTF-positive neurons and immunofluorescence in the dorsal sensory columns. This treatment evidently improved the tissue cytoarchitecture, especially when compared to the untreated SCI experimental group (Fig. 8I). Surprisingly, the combined treatment of GnRH and GH nearly eliminated the positive immunostaining (Fig. 8J) but had a clear beneficial effect on maintaining tissue integrity and organization.

### Effect of GnRH and GH on PSD95 and synaptophysin immunoreactivity in SCI

**Fig. 9:**
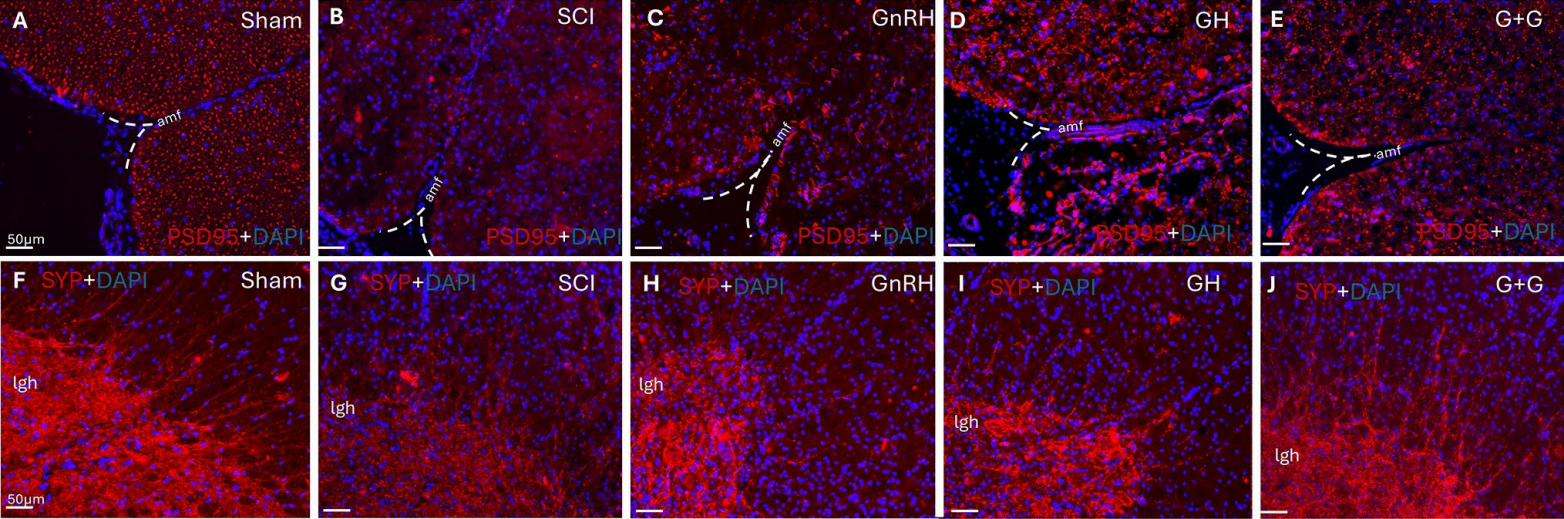
PSD95 and SYP immunohistochemistry in spinal cord tissue. Transverse sections of spinal cord collected from distal side of the injury (T11-T12). PSD95-immunofluorescence (red) at 10 × magnification (**A-E**) and SYP-immunofluorescence (red) at 10 × magnification (**F-J**). DAPI was used for nuclei counterstaining (blue). Arrows show representative immunoreactive cells. Experimental groups: sham (control), spinal cord injury (SCI), GnRH, GH and GnRH + GH (G + G). **amf**: anterior medial fissure**; lgh**: lateral grey horn. Scale bar (50 µm).

The presence of postsynaptic density protein 95 (PSD95) and synaptophysin (SYP) immunoreactivities were analyzed by IHC in transverse sections of the caudal spinal cord, with the SCI epicenter at T10 serving as the anatomical reference. In the control group, PSD95-IR was moderate and uniformly distributed throughout the spinal cord tissue (Fig. 9A). In contrast, lesioned animals without treatment exhibited an almost complete absence of PSD95-IR (Fig. 9B). GnRH treatment led to an increase in PSD95 immunofluorescence, particularly in the white matter adjacent to the anterior median fissure (Fig. 9C). GH treatment also resulted in a marked upregulation of PSD95-IR, although the pattern appeared disorganized (Fig. 9D). The combination treatment group (G + G) demonstrated a PSD95-IR pattern similar to that of the sham control. However, fewer cells displayed positive PSD95 immunofluorescence staining in the combined group (Fig. 9E).

On the other hand, SYP immunostaining was consistently observed in the gray matter, with some radial projections extending into the white matter in the lateral horn area (lgh) in the control group (Fig. 9F). In the SCI group, there was a marked decrease in SYP immunofluorescence (Fig. 9E). However, this reduction was similarly reversed by independent treatments with either GnRH (Fig. 9H) or GH (Fig. 9I). Notably, the combination treatment (G + G) resulted in SYP-IR comparable to that of the sham control in the gray matter, and showed a great number of projections into the white matter in the lgh region (Fig. 9J).

#### Effect of GnRH and GH upon BBB scale and ankle kinematics

**Fig. 10:**
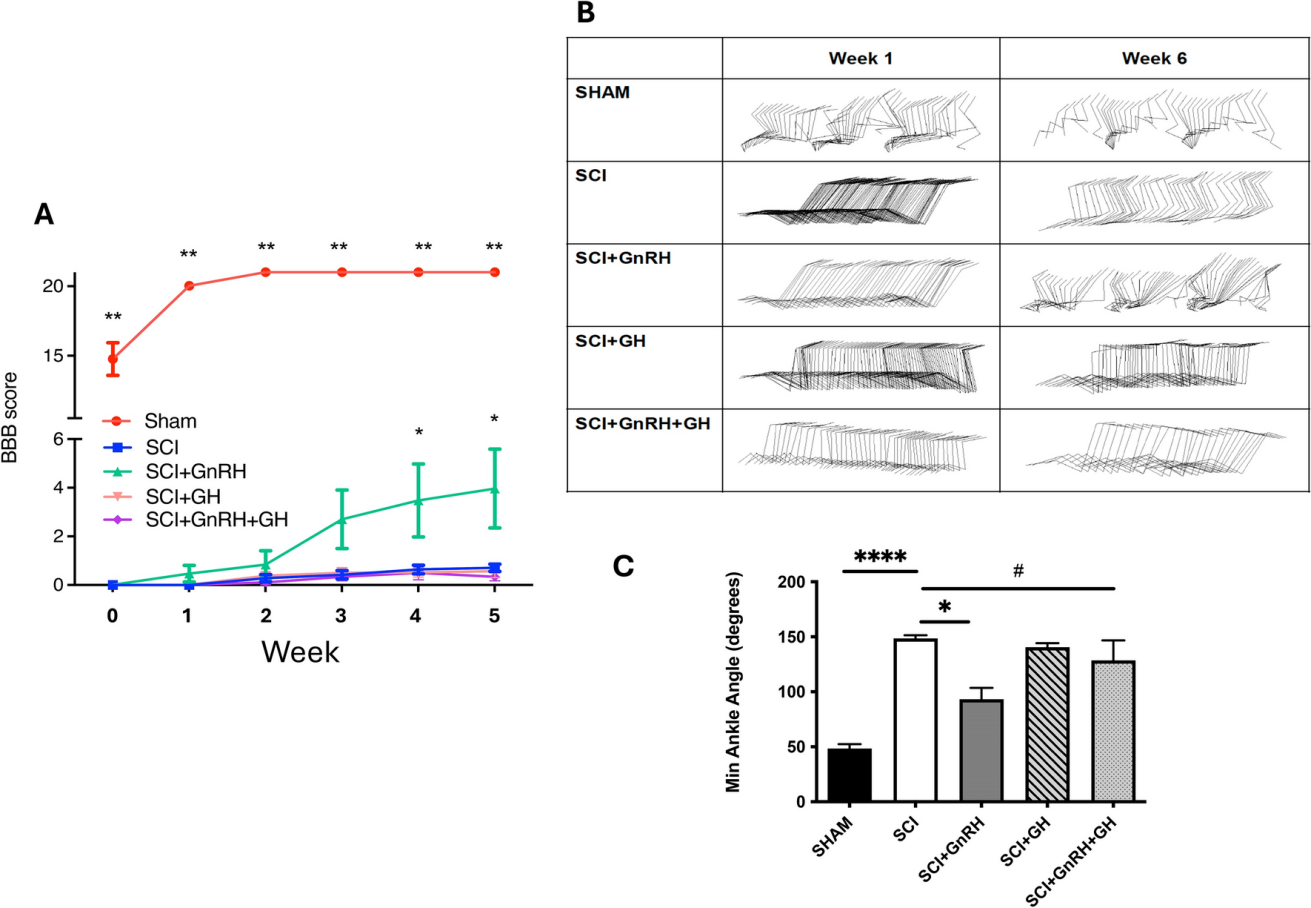
Effect of GnRH and/or GH treatments on locomotor activity of the hindlimbs after SCI graded on BBB scale. (**A**) Data points represent mean ± SEM of 10 animals per group. Sham group (red), SCI group (blue), GnRH group (green), GH group (pink), GnRH + GH group (purple). BBB values in a range from 0 (lowest) and 21  (maximal). (**B**) Graphical representation of gait kinematics at 0 and 6 weeks post-SCI. (**C**) Minimal ankle angle measured at week 4 post injury; units in degrees. One-way Brown-Forsythe and Welch ANOVA with Fisher´s LSD *post-hoc* test recognized significant differences. A p-value under 0.05 indicated statistical significance and is represented with asterisks as follow: ^#^,* P < 0.05, ** P < 0.01 and **** P < 0.0001.

The BBB locomotor rating scale was used to assess the deleterious motor consequences of SCI in rats (Fig. 10A). The sensitivity of this rating scale can differentiate the locomotor abilities of the hindlimbs. The scale has a total score of 21, where 0 represents total paralysis of the hind legs and 21 represents normal walking. To perform this, the rats were placed in an area bounded by transparent walls and allowed to roam freely. The position of their hind legs, trunk, and tail were assessed, as well as their ability to bear weight. The movement of the hindlimbs is graphically represented in Fig. 10B, showing important differences among groups at 0 and 5 weeks after SCI. The evaluation was carried out by 3 different independent observers and the average of the 3 scores was recorded to avoid observer error. The results indicated that, at week 0, rats in the sham group had a mild impairment in the movement of their hind legs as result of non-damaging surgery, with an average score of 14.7 on the scale (red; Fig. 10A). However, this group already achieved total motor recovery by week 2, where they averaged the highest score on the scale (21), that is, normal walking. In contrast, the lesioned group showed a very strong deficiency in their hindlimb motor ability with a score of 0 after the first 2 weeks which remained low until the end of the experiment at week 5 (score 0.8). Interestingly, the group of rats treated with GnRH showed a tendency to improve starting at week 1, reaching a statistically significant improvement in weeks 4 and 5 when compared against the SCI group at the same time (green; Fig. 10A). On the other hand, the injured groups treated with either GH (pink; Fig. 10A) or the combined hormone treatment (GnRH and GH; purple) showed a slight increase in score from week 2, but without significant changes in comparison to the SCI group. In addition, ankle angle was quantified for motion analysis, showing an angle of 50° in the control group (Fig. 10C). This angle was significantly increased following SCI (P < 0.0001). Treatment with GnRH significantly reduced the ankle angle (P < 0.05), while GH alone had no effect. However, the combination of both hormones in the G + G group resulted in a slight, but significant, reduction in ankle angle compared to the SCI group (P < 0.05). Videos recording motion activity demonstrate a clear and significant deleterious effect on rat hindlimbs at 5 weeks post-SCI. On the other hand, GnRH treatment showed a strong effect on recovery motion, which was abolished when combined with GH, in the G + G group. (*videos provided in the following link*): https://drive.google.com/drive/folders/1SZluVoWZROaAxfsDAY1hySB4a4ZZcd_-?usp=share_link

## Discussion

This study provides evidence demonstrating the neurotrophic effects of two hormones with well-established canonical actions: GnRH, known for its neuroendocrine control of reproduction, and GH, recognized for its role in musculoskeletal growth, in a model of neural damage in the rat spinal cord. Our research revealed that chronic administration of GnRH and/or GH over 5 weeks induced recovery in the mRNA expression of several classical neurotrophins and various markers associated with synaptic function in the spinal cord after using a mechanical compression model via catheter inflation at the T10 level in ovariectomized (OVX) rats. As anticipated, spinal cord injury significantly reduced the transcriptional rate of most genes related to synaptic restoration in the damaged area. It is noteworthy that the combined treatment of GnRH and GH most significantly increased messenger RNA levels for synaptic function proteins, including SNARE complex proteins, synaptic markers, and neurotrophins.

NGF function is primarily associated with the survival, maintenance, and development of the central nervous system^24,25^; however, in its precursor form, pro-NGF can induce cell death via apoptosis^26,27^. In this study, we found that NGF expression levels significantly increased in the injury epicenter, correlating positively with neural damage specifically in this area. Conversely, in the caudal end, NGF, alongside its counterparts BDNF and NT3, exhibited consistent gene transcription recovery. We previously reported that GH can increase BDNF synthesis under various neural damage conditions, such as hypoxia and excitotoxicity^28^. Here, we found that the combination of GnRH and GH elicited the strongest response across all studied dorsal spine sections. There was a diminished response to hormonal treatments, whether alone or in combination, at the damage epicenter area for BDNF and NT3 genes. However, the increase in NGF mRNA in this section was significantly inhibited in the G + G group and only attenuated by individual hormonal treatments. In a separate experiment, tissue samples were morphologically analyzed with specific antibodies for NT3, clearly identifying large motoneurons in the ventral horns of the gray matter with intense, well-defined immunoreactivity delineating the soma size. These results clearly implicate a complex network of neurotrophic factors that dynamically respond to damage and treatments.

Expression levels of neuroligins and neurexins, accepted as neural injury recovery markers, are specifically expressed in functional synapses, allowing for mRNA quantification as an indirect measure of their production and functionality^29^. We observed that our treatments partially restored the expression of Nlgn-1 and Nxn1, particularly at 3 weeks post-thoracic SCI. SNARE complex proteins are regulators of intracellular transport, especially of neurosecretory vesicles, determining their destination and regulating the specificity of neuropeptide and neurotransmitter secretion^30^. Conclusive evidence was found on the effects of GnRH and GH in this intracellular transport system, and their neurosecretory and synaptic function. The combination was able to increase the expression of SNAP25, SYP, and syntaxin-1, more so than individual treatments, which primarily responded at the caudal end of the collected tissue. The injury area was particularly susceptible to hormonal stimulation, recording significant increases in SNAP25 mRNA expression, indicating high secretory activity in the neurons induced by treatments as part of the tissue recovery process, this is important since it plays a crucial role in sensory functional recovery following a SCI in rats with spinal cord transection^31^. In the caudal part, GnRH had no significant effect on the expression of SYP, SNAP25, and syntaxin-1 genes compared to the damaged group. Interestingly, GH had a restorative effect on the genetic expression of all three SNARE activity markers, and this effect was moderately increased when administered in combination with GnRH compared to GH alone. We also analyzed the presence of PSD95 and SYP by IHC, as markers for synaptic damage recovery. It was found that, for both proteins, SCI induced a significant decrease in their immunoreactivity, but it was partially restored by the individual or combined treatments with the hormones. The observed effect of GnRH and/or GH on these synaptic and myelin recovery markers showed a positive outcome only for GnRH in locomotion. However, in all cases, there was a partial or nearly complete recovery in protein presence and tissue organization. This suggests that other mechanisms, such as intracellular signaling and autocrine/paracrine interactions, are involved in the observed neurophysiological antagonism, which deserves further investigation.

Based on evidence, we observed that GH, GnRH and the combined G + G treatments clearly increased the amount of myelin sheath stained by the Klüver-Barrera method and also MBP immunoreactivity in comparison to the lesioned group. Additionally, the cellular organization of the tissue observed in longitudinal sections showed disorganized recovery patterns and the presence of fibrillar structures positive for III-β-tubulin in random arrangements as observed by immunofluorescence (Sup. Figure 2C). Further studies are required to evaluate the optimal doses of GH and treatment administration protocols to determine if potential overstimulation of GH induces aberrant motor reconnection, impeding improvement in walking kinematics. It is documented that excess GH can lead to receptor saturation and/or desensitization^32^. Literature reports suggest that GH receptor is not present in motoneurons in the ventral horns in the thoracic area, which should be considered as a possibility for the lack of motor recovery^33^. However, there are reports demonstrating that GH combined with rehabilitation improves motor score, quality of life and sensory perception in patients with SCI^34–36^. In addition, ghrelin, a potent GH-secretagogue is able to exert antiapoptotic effects and improves functional recovery after SCI, most likely involving GH pituitary release^37^. This inhibitory interaction is currently under investigation in different damage models in our laboratory. We have preliminary data suggesting that chronic administration of GH over 3 to 5 weeks under neural damage conditions results in interference with the neurotrophic effect, which has been extensively documented individually for both peptide messengers^10^. Our myelin sheath integrity analysis yielded interesting information regarding abnormal production of myelin in the acute and subacute phases of the damaged spinal cord as previously reported^38^. In the control group without compressive damage, very intense staining, and the parallel arrangement of myelin fibers in the white matter were observed, which was completely lost in the lesioned group. The cephalic end of the spinal tissue maintained its organization best after the insult, where GH had the greatest effect in positively marked areas. However, aberrant myelin deposits were observed in the GH and combined treatment groups. These results were also supported by the effects observed upon MBP-IR with the treatments. It is noteworthy that myelin and its debris have been reported to play an antagonistic role in axon regeneration following a spinal cord injury^39^. A marked increase in myelin marking in the caudal part with the combination of both hormones can partially explain the motor recovery impairment.

CNTF is currently one of the most studied neurotrophic factors as a candidate for administration in various CNS pathologies, as it is in advanced phases in various clinical trials for its application as neuroprotective therapy^40–42^. In this study, we identified CNTF-positive motoneurons distributed throughout the gray matter with positive immunofluorescence, where sections from animals treated with GnRH were more similar to the control. CNTF has been previously identified in motoneurons during rat development and has well-accepted roles in neural protection/regeneration^43^. Our results showed that the combinatory treatment abolished CNTF-IR in tissue slides from the caudal end of the damaged spinal cord and has positive correlation with the locomotive recovery impairment.

Using the same SCI model, we recently reported sensory recovery induced by both GnRH and GH post-SCI in the hot plate assay, correlating directly with a decrease in inflammatory markers and glial activity expression^11^. Our hypothesis postulated that GH and GnRH, alone or in combination, could improve walking recovery, assessed through kinematic analysis to obtain the BBB scale. Unexpectedly, we found that only GnRH had significant effects on the partial recovery of hindlimb functionality, and simultaneous application of both hormones blocked neuromotor recovery. However, it is important to note that our findings align perfectly with data from previous experiments using leuprolide acetate, a GnRH analog, and synthetic peptide administration, documenting a similar recovery pattern on the BBB scale as this study, which has been a world-wide accepted score for SCI recovery^13^. In addition, we also observed a significant ankle angle recovery induced by GnRH but not by GH.

In the pituitary gland, activation of the GnRHR upregulates the synthesis and release of follicle-stimulating hormone (FSH) and luteinizing hormone (LH) through a complex network of intracellular pathways that includes its canonical G-protein coupled signaling^44^. During this process, the activation of GnRHR includes the inset of Gq/11 proteins, which subsequently activate phospholipase C (PLC). PLC generates diacylglycerol (DAG) and inositol 1,4,5-trisphosphate (IP3)^45^. DAG activates the protein kinase C (PKC) pathway, while IP3 stimulates the release of intracellular calcium, further amplifying the signaling cascade and vesicular release^45^. Additionally, the MAPK/ERK, PKA, and Akt pathways also play significant roles during gonadotropin synthesis and release. In extrapituitary tissues, such as the CNS, these pathways are involved in cell survival, neural function, and neurotrophic actions^46^. Evidence suggests that GnRH and its analog, leuprolide acetate, have regenerative and protective properties in various models of neural damage, and they could be acting through the same molecular mechanisms^13,47–49^. The GnRH beneficial effects have been demonstrated both in experimental animal models and in clinical trials, evidencing their therapeutic potential for paralyzed​ patients^13,46,50,51^. However, despite these promising outcomes, the molecular mechanisms and interactions underlying the effect of GnRH, or its analogs in the nervous system, remain largely unexplored.

On the other hand, it is now recognized that GH binding to GHR activates multiple signaling pathways that play significant roles in neuroprotection within the central nervous system (CNS)^52^. The intracellular domain of GHR is coupled to its canonical JAK-STAT signaling pathway^53^. However, upon GHR activation, the PI3K/Akt, MAPK-ERK, and Notch signaling pathways are also triggered^54^. Axonal growth and regeneration are regulated by intrinsic and extrinsic factors, with various transcription factors, such as the KLF family, maintaining a balance of gene expression and repression^55^. GH impacts these KLF transcription factors through the JAK-STAT pathway, leading to changes in histone acetylation and promoting axonal regeneration^56^. Specifically, STAT5 and STAT3 are associated with the activation of genes directly related to neuronal survival, axonal regeneration, and synaptogenesis^57^. Additionally, IGF-1, NT3, CNTF, and BDNF are upregulated by GHR activation^11^. These neurotrophins are responsible for maintaining neural tissue homeostasis and are also associated with neuroprotective and regenerative processes. In addition, it is well demonstrated that GH induces strong anti-apoptotic effects during CNS development and neuroprotection, mediated through classical Bcl-2 family proteins in the chicken retina^54,58,59^.

Neuroinflammation plays a critical role following spinal cord injury (SCI), with interactions between resident glial cells and infiltrating immune cells significantly impacting recovery. GnRH has demonstrated notable anti-inflammatory effects in the nervous system^11,60^. For instance, in a rat model of experimental autoimmune encephalomyelitis (EAE), GnRH administration significantly reduced NF-κB activation during disease progression and downregulated mRNA expression levels of the proinflammatory cytokines IL-1β, IL-17A, and TNF-α during the recovery phase^60^. These effects were correlated with a decrease in the severity of locomotor deficits induced by the treatment. Moreover, GnRH has been shown to ameliorate amyloid β-induced cognitive decline, potentially through the local production of neurosteroids^61^. Our group recently reported that both GH and GnRH can reduce the expression of proinflammatory markers (IL-6, IL-1β, and iNOS) as well as glial activity markers (Iba1, CD86, CD206, vimentin, and GFAP) in spinal cord tissue, thereby enhancing sensory recovery in injured animals^11^. For GH, the anti-inflammatory effects likely involve the NF-κB signaling pathway, as GH has been observed to reduce NF-κB activity in macrophages^62–64^. These macrophages infiltrate the injury site and interact with astrocytes and neurons during glial scar formation and critically define the recovery possibilities^65^.

In summary, this study identified that both GnRH and GH are capable to restore the expression of synaptic function markers, neurovesicular transport, and locally expressed neurotrophins in the damaged area. Interestingly, only GnRH treatment was capable of restoring hindlimb function, likely due to an aberrant regenerative effect induced by GH. GnRH improved tissue organization and restored immunoreactivity to NT3 and CNTF in ventral horn motoneurons. In addition, myelination as well as SYP and PSD95 synaptic markers decline provoked by the lesion was partially reversed with the treatments. The potential use of GnRH and/or GH requires more detailed research, particularly regarding the observed antagonism in motor recovery when applied in combination.

## Materials and methods

### Animals

Female Wistar rats (approximately 250 g) were employed in the study. These animals, bred at the Institute of Neurobiology (INb)-UNAM, were moved to the Universidad Autónoma de Aguascalientes (UAA) vivarium for acclimation to a 12 h-light/12 h-dark cycle and temperature between 20–22 °C. During their stay, they had unrestricted access to Purina chow pellets and tap water. The studies were in alignment with the bioethical norms of the National Health and Medical Research Council. Also, both the UAA and INb-UNAM (protocol 122A) animal ethics committees endorsed the research methods. All procedures adhered to the animal welfare regulations of the UAA (CEADI/UAA/0025/18) and to the ARRIVE guidelines.

### Experimental design

Utilizing G*Power 3.1 software, we achieved an effect size of d = 0.55, which ranges from moderate to large. This was done under the conditions of 5% type-I error rate (alpha: 0.05) and a power of 80% (β = 0.2) across five experimental groups, each containing eight animals. Furthermore, to accommodate potential losses such as injury-related fatalities or bioethical euthanasia, we included an additional four animals per group. Our initial number or rats per group was 12, which is a reliable sample size. The rats were divided randomly into five groups: a) Sham SCI surgery (sham group), b) SCI with physiological saline (SCI group), c) SCI with GnRH treatment (GnRH group), d) SCI with GH treatment (GH group) and e) SCI treated with both GnRH and GH (G + G group). In the sham group, the standard surgical procedure was performed, excluding the catheter insufflation. The treatment regimen consisted of a daily subcutaneous injection of 0.15 mg/kg human recombinant growth hormone (rhGH) (somatropin; Genotropin C, Pfizer, México)^11^and/or human recombinant gonadotropin-releasing hormone (GnRH, 0.06 mg/kg) (L7134, Sigma, MO, USA) given intramuscularly every 12 h, as described elsewhere^66,67^. Spinal tissues were preserved at -70 °C until examination. Figure 1A shows the experimental protocol in a graphical version.

### Spinal cord injury surgery and postoperative care

Rats were ovariectomized (OVX) one week prior to the study due to the estrogen secretion effects of GnRH, which could serve as a neuroprotector, potentially influencing the study’s results^46^. A week after recovery from ovariectomy, rats underwent SCI via balloon compression, with methodology adapted from Vanicky et al^68^. and previously described^11–13^. Rats exhibiting complete paraplegia post-surgery were included in the study. Following the operation, the subjects received daily injections of penicillin (Penprocilin; 5000 IU; IM) for a week to ward off infections and metamizole (15 mg/kg; IM) for three days for pain relief. Their urinary bladders were manually emptied twice daily until bladder reflex restoration. Rats displaying chronic stress, acute pain, fever, or infection were euthanized according to bioethical guidelines.

### Gene-expression quantification by real-time polymerase chain reaction (qPCR)

At 3 weeks post-injury, rats were euthanized and perfused with saline solution under pentobarbital anesthesia. Tissue samples were immediately frozen and stored at -70 °C. The Zymo Direct-zol purification kit and TRIzol (Zymo Research Corp., Irvine, CA, USA) were employed to extract RNA from spinal cord tissue lysates. DNAase I (Promega) was used for DNA digestion, and cDNA was synthesized from the extracted RNA. The qPCR procedures and conditions were precisely as those mentioned in Martinez-Moreno et al^11,18^.. Oligonucleotides are shown in Table 1.

**Table 1:** Oligonucleotides.

Target gene	Primer	Sequence	Size	Accession#
BDNF	Fwd	TCCACCAGGTGAGAAGAGTGATG	159 bp	NM_001270638.1
	Rev	TCACGCTCTCCAGAGTCCCATG		
NGF	Fwd	CCCCGAATCCTGTAGAGA	149 bp	NM_001277055.1
	Rev	CACGCAGGCTGTATCTAT		
Ntf3	Fwd	AACGAGGTGTAAAGAAGC	155 bp	NM_001270870.1
	Rev	TGTCTATTCGTATCCAGC		
Nlgn1	Fwd	GCAAGACCAGAGTGAAGACTGT	128 bp	NM_053868.2
	Rev	TGTCCCGAATATCTCCTTTTCTAC		
Nxn1	Fwd	TGGACTTGAATGGCAGGCTT	100 bp	NM_021767.3
	Rev	TCTTGACAGGTTGTGCTGGG		
SNAP25	Fwd	CATGGGCAATGAGATTGACA	120 bp	NM_001270576.1
	Rev	CCACTTCCCAGCATCTTTGT		
Synaptophysin	Fwd	GTGCCAACAAGACGGAGAGT	115 bp	NM_012664.3
	Rev	ATCTTGGTAGTGCCCCCTTT		
Syntaxin	Fwd	TGGACTCCAGCATCTCGAAG	191 bp	NM_053788.3
	Rev	CCTCTCCACGTAGTCCACAG		
RPS18	Fwd	TTCAGCACATCCTGCGAGTA	136 bp	NM_213557.1
	Rev	TTGGTGAGGTCAATGTCTGC		

### Immunohistochemistry & fluorescence microscopy

Briefly, at five weeks post-SCI, rats were euthanized and perfused with ice-cold saline solution (sodium chloride at 0.9%; Pisa Flexoval CS). Their spinal cords were carefully extracted by injecting water in one side of the vertebrae canal. Sections were labeled as: a) cephalic (T7-T8), b) injury site (T9-T10), and c) caudal (T11-T12). The tissue processing, fixation, mounting, and image capture methods were performed as detailed previously^11^. For immunohistochemical analysis, primary polyclonal antibodies (Table 2) and secondary antibodies conjugated with Alexa Fluor fluorophores were used.

**Table 2:** Antibodies.

Target	Host/Type	Dilution	Source	Cat.No
NT-3	Rabbit/polyclonal	1:300	Abcam	Ab53685
PSD95	Rabbit/polyclonal	1:200	Invitrogene	51–6900
MBP	Rabbit/polyclonal	1:500	Invitrogen	A5-78,397
SYP	Rabbit/polyclonal	1:200	Millipore	AB9272
III β-tubulin	Mouse/monoclonal	1:500	Abcam	AB78078
CNTF	Rabbit/polyclonal	1:500	Abcam	AB46172
Mouse IgG	Rabbit/Alexafluor488	1:1000	Invitrogen	A-11029
Rabbit IgG	Goat/Alexafluor594	1:1000	Invitrogen	A-11012

### BBB scale for locomotor recovery

Locomotive function was assessed once a week after surgery using the BBB scale^12^. This scale rates the animal motor performance from 0 to 21, with 0 indicating no movement and 21 representing typical movement, stability, and coordination. During these evaluations, three impartial observers, unaware of the treatment each rat received, recorded the scores. Gait kinematics analysis was performed weekly for 5 weeks starting the day after surgery. The points corresponding to the hip, knee, ankle joints and the head of the fifth metatarsal were marked on the rat’s skin. Subsequently, the animals were introduced individually onto a walkway with transparent walls and their movements were recorded with a video camera (GoPro HERO5 Black, San Mateo, CA, USA) at a speed of 60 frames per second. To obtain an image of the kinematics of the animals’ hind legs in three continuous strides, the video segment in which the animal walked 5 continuous steps without distraction or stopping was selected. The Tracker free video software (Open-Source Physics) was used to digitize the movements of the points marked on the previously described joints and thus obtain a graphical image of the sequence of 3 continuous strides.

### Kinematic analysis

Ankle kinematics were carried out weekly for 5 weeks starting next day after surgery. Points corresponding to ankle angle (the knee joint, the lateral malleolus and fifth metatarsal head) were marked on the rat skin. The same operator performed all marker placements to avoid inter-tester variability. Subsequently, the animals were each introduced separately, a runway with transparent acrylic walls, where their movement was recorded with a commercial video camera (GoPro HERO5 Black, San Mateo, CA, USA) at a rate of 60 images per second. For kinematic gait analysis it was selected the video segment in which the animal walked 3 continuous steps without any distraction or detention. The study was limited to bi-dimensional analysis of the left hindlimb of the animal. Tracker video analysis free software was used to digitize the movement of the markers and obtain the data of interest. Numerical analysis was performed with GraphPad Prism version 6.00 for Mac (GraphPad Software, San Diego, CA, USA).

### Statistical Analysis

Values are represented as mean ± SEM. One-way Brown-Forsythe and Welch ANOVA with Fisher´s LSD post-hoc test identified significant differences. A p-value under 0.05 indicated statistical significance and is represented with asterisks as follow: * P < 0.05, ** P < 0.01, *** P < 0.001 and **** P < 0.0001. Outliers were detected using the ROUT method (Q = 1%) in Prism Graph 9 (GraphPad, San Diego, CA, USA). Sample size variability in the analyzed groups ranged from 5 to 10 OVX-rats due bioethical consideration since animals with infection, fever, stress, evident pain, or abnormal behavior were euthanized. Data was derived from two separate experiments and values were considered for statistical analysis without pseudo-replication.

## Supplementary Information

Below is the link to the electronic supplementary material.

Supplementary Information 1

Supplementary Information 2.

Supplementary Information 3.

## Data Availability

The datasets generated during and/or analyzed during the current study are available from the corresponding authors on reasonable request.
