# Supplementary material for: Neurotrophic and synaptic effects of GnRH and/or GH upon motor function after spinal cord injury in rats
Source: Sci Rep. 2024 Nov 2;14:26420. doi: 10.1038/s41598-024-78073-3 (PMC11531546; doi:10.1038/s41598-024-78073-3)
Supplement: Supplementary file 1 — Supplementary Information 1 [file 41598_2024_78073_MOESM1_ESM.pdf]

Sup. Figure 1

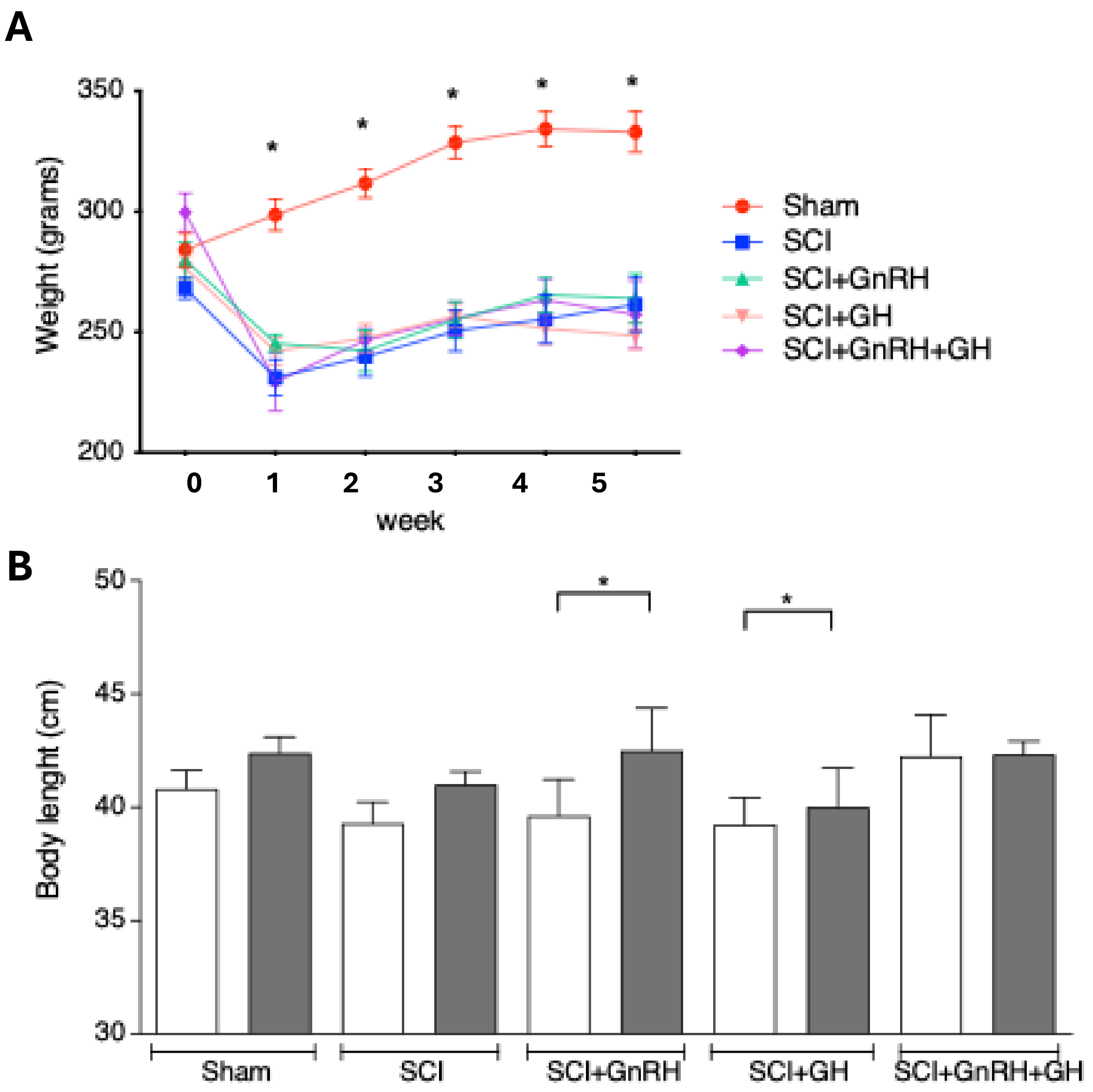

A
350
300
250
200
Weight (grams)
0
1
2
3
4
5
week
Sham
SCI
SCI+GnRH
SCI+GH
SCI+GnRH+GH
B
50
45
40
35
30
Body lenght (cm)
Start
End point
Sham
SCI
SCI+GnRH
SCI+GH
SCI+GnRH+GH

Sup. Figure 2

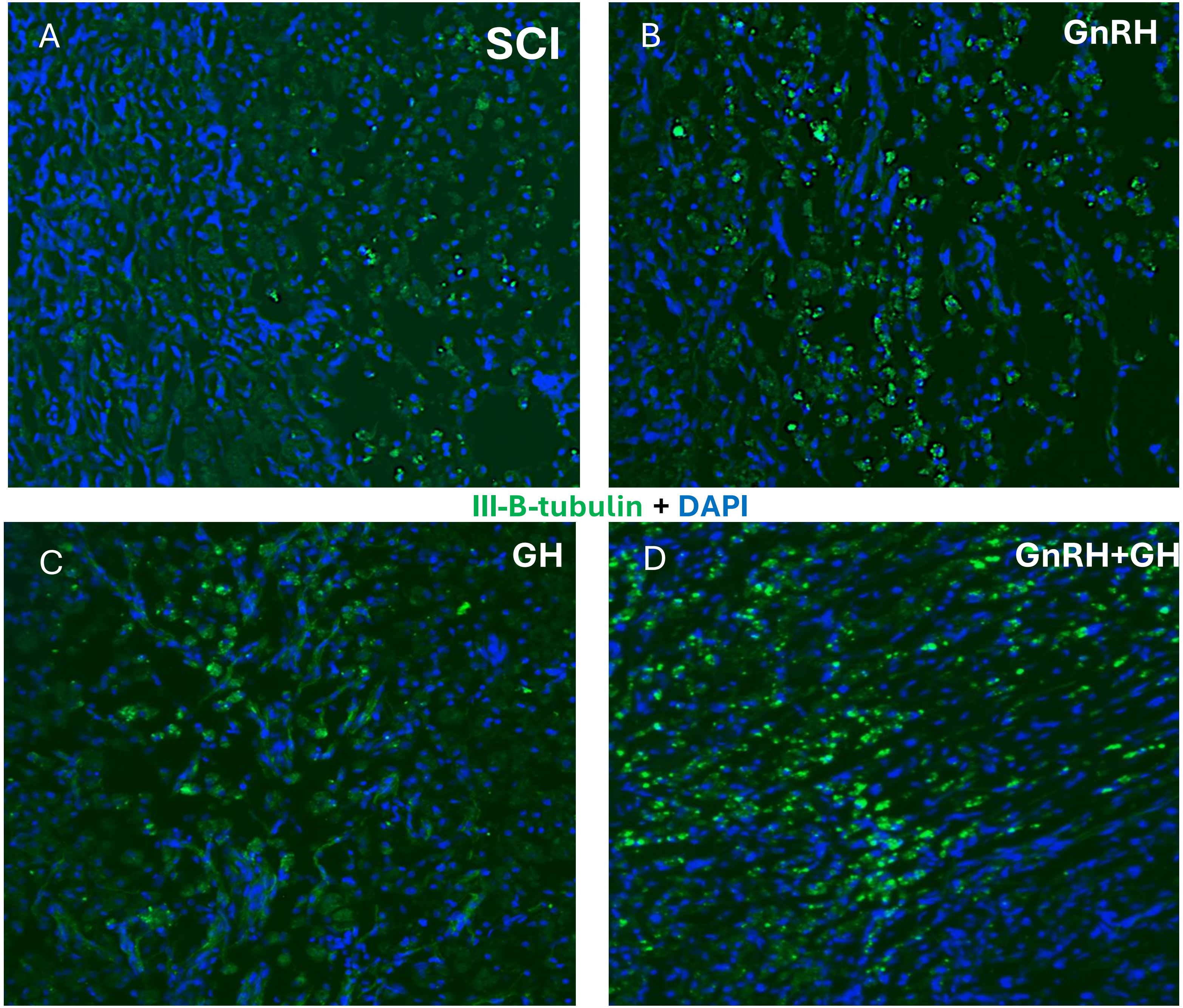

Supplementary Information

Figure Legends for Supplementary Figures

Fig. Sup. 1. Weight and length of OVX rats. **(A)** Shows the effect of SCI, GnRH and/or GH treatments during 6 weeks on body weight. **(B)** Show the effect of treatments upon body length. Units in grams and centimeters.

Fig. Sup. 2. III-beta-tubulin immunoreactivity in spinal cord tissue collected from the injury epicenter. Longitudinal sections of spinal cord collected from epicenter of the injury (T10). Experimental groups: (**A**) spinal cord injury without treatment (SCI), (**B**) GnRH, (**C**) GH, and (**D**) GnRH + GH: Green, III-B-tubulin immunofluorescence; Blue: DAPI staining (nuclei).
