# Supplementary material for: Neurotrophic and synaptic effects of GnRH and/or GH upon motor function after spinal cord injury in rats
Source: Sci Rep. 2024 Nov 2;14:26420. doi: 10.1038/s41598-024-78073-3 (PMC11531546; doi:10.1038/s41598-024-78073-3)
Supplement: Supplementary file 2 — Supplementary Information 2. [file 41598_2024_78073_MOESM2_ESM.docx]

High Resolution Figures (zip file)

Download link:

<https://drive.google.com/file/d/1muSXYuOKl13xbRCk4WKjApkrL209E1b2/view?usp=sharing>
