# Supplementary material for: Neurotrophic and synaptic effects of GnRH and/or GH upon motor function after spinal cord injury in rats
Source: Sci Rep. 2024 Nov 2;14:26420. doi: 10.1038/s41598-024-78073-3 (PMC11531546; doi:10.1038/s41598-024-78073-3)
Supplement: Supplementary file 3 — Supplementary Information 3. [file 41598_2024_78073_MOESM3_ESM.docx]

Kinematics Videos

<https://drive.google.com/drive/folders/1SZluVoWZROaAxfsDAY1hySB4a4ZZcd_-?usp=share_link>
